# Projections of nucleus accumbens adenosine A_2A_ receptor neurons in the mouse brain and their implications in mediating sleep-wake regulation

**DOI:** 10.3389/fnana.2013.00043

**Published:** 2013-12-10

**Authors:** Jian-Ping Zhang, Qi Xu, Xiang-Shan Yuan, Yoan Cherasse, Serge N. Schiffmann, Alban de Kerchove d’Exaerde, Wei-Min Qu, Yoshihiro Urade, Michael Lazarus, Zhi-Li Huang, Rui-Xi Li

**Affiliations:** ^1^Department of Anatomy, Histology and Embryology, Shanghai Medical College, Fudan UniversityShanghai, China; ^2^Department of Pharmacology, State Key Laboratory of Medical Neurobiology, Institute of Brain Sciences, Shanghai Medical College, Fudan UniversityShanghai, China; ^3^Department of Molecular Behavioral Biology, Osaka Bioscience InstituteSuita, Osaka, Japan; ^4^International Institute for Integrative Sleep Medicine, University of TsukubaTsukuba, Ibaraki, Japan; ^5^Laboratory of Neurophysiology, Université Libre de Bruxelles, ULB Neuroscience InstituteBrussels, Belgium

**Keywords:** nucleus accumbens, adeno-associated virus, Cre-Lox, green fluorescent protein, mouse, sleep

## Abstract

Adenosine A_2A_ receptors (A_2A_Rs) in the nucleus accumbens (Acb) have been demonstrated to play an important role in the arousal effect of adenosine receptor antagonist caffeine, and may be involved in physiological sleep. To better understand the functions of these receptors in sleep, projections of A_2A_R neurons were mapped utilizing adeno-associated virus (AAV) encoding humanized Renilla green fluorescent protein (hrGFP) as a tracer for long axonal pathways. The Cre-dependent AAV was injected into the core (AcbC) and shell (AcbSh) of the Acb in A_2A_R-Cre mice. Immunohistochemistry was then used to visualize hrGFP, highlighting the perikarya of the A_2A_R neurons in the injection sites, and their axons in projection regions. The data revealed that A_2A_R neurons exhibit medium-sized and either round or elliptic perikarya with their processes within the Acb. Moreover, the projections from the Acb distributed to nuclei in the forebrain, diencephalon, and brainstem. In the forebrain, A_2A_R neurons from all Acb sub-regions jointly projected to the ventral pallidum, the nucleus of the diagonal band, and the substantia innominata. Heavy projections from the AcbC and the ventral AcbSh, and weaker projections from the medial AcbSh, were observed in the lateral hypothalamus and lateral preoptic area. In the brainstem, the Acb projections were found in the ventral tegmental area, while AcbC and ventral AcbSh also projected to the median raphe nucleus, the dorsal raphe nucleus, and the ventrolateral periaqueductal gray. The results supply a solid base for understanding the roles of the A_2A_R and A_2A_R neurons in the Acb, especially in the regulation of sleep.

## INTRODUCTION

The adenosine A_2A_receptors (A_2A_Rs) in the nucleus accumbens (Acb), that consists of the core and shell sub-regions, play a critical role in many important physiological and pathological processes, including sleep, feeding, locomotion, motivation, and addiction (Barraco et al., 1993, 1994; Nagel et al., 2003; Kelley, 2004; Huang et al., 2007, 2011; Mingote et al., 2008; Lazarus et al., 2011, 2012, 2013; O’Neill et al., 2012). However, the cellular morphology and projection patterns of Acb have been characterized based mainly on traditional tracing techniques with nonspecific tracers (Heimer et al., 1991; Meredith et al., 1992; Zahm and Heimer, 1993; Jongen-Rêlo et al., 1994; Goto and Grace, 2008), which is insufficient to explain the role of the adenosine A_2A_R in the Acb. The A_2A_R-expressing neurons and their related neural pathways execute the specific actions triggered by the type of receptor, and so specifically analyzing the neural pathway is critical to fully understand the function of the receptor. Because of this, identifying the projections of A_2A_R neurons has become an important issue (Lazarus et al., 2012).

Because of limitations in tracing techniques, little data are available describing the efferent projections of A_2A_R neurons in the Acb. Those data that are available have been obtained using conventional tracers, such as autoradiographic fiber-tracing (Nauta et al., 1978), wheat germ agglutinin conjugated to horseradish peroxidase (WGA-HRP; Heimer et al., 1991), phaseolus vulgaris-leucoagglutinin (PHA-L; Zahm and Heimer, 1993), and biotinylated dextran amine (BDA; Usuda et al., 1998). These studies report that Acb targets the ventral pallidum, lateral hypothalamus, ventral tegmental area, substantia nigra pars compacta, and the pedunculopontine tegmental nucleus (Heimer et al., 1991; Zahm and Heimer, 1993; Usuda et al., 1998). Neurons in the Acb are divided into the GABAergic projection neurons and interneurons (Ferré et al., 2007; Schiffmann et al., 2007), and GABAergic projection neurons, which can be further sub-characterized into enkephalinergic and dynorphinergic neurons. Adenosine A_2A_Rs are predominantly localized in the GABAergic enkephalinergic neurons and the glutamatergic terminals in the Acb (Ferré et al., 2007; Schiffmann et al., 2007). The efferent regions of Acb neurons therefore do not stand for projection sites of A_2A_R neurons in Acb.

The approaches based on Cre-LoxP technology have been used for the neural tracing of selected neurons, whereby an adeno-associated virus (AAV) is carrying a humanized Renilla green flu-orescent protein (hrGFP) that is transcriptionally silenced by a neo cassette flanked by LoxH/LoxP sites (Gautron et al., 2010), but can be activated as a tracer in the presence of Cre recombinase. The AAV is stereotaxically injected into a brain that expresses Cre under the control of the adenosine A_2A_R promoter, resulting in robust hrGFP expressions specifically in A_2A_R neurons (Durieux et al., 2009, 2012) Moreover, in these studies hrGFP was efficiently transported to brain regions that were innervated by A_2A_R neurons.

Using the techniques mentioned above, we stereotaxically injected the AAV into the core (AcbC) and shell (AcbSh) of the nucleus accumbens. We then used immunohistochemical and/or immunofluorescence techniques to detect the expression of the A_2A_R neurons in the injection sites and their axon distributions in various brain regions after 4 weeks of injections (Chamberlin et al., 1998). Special emphasis was placed on well-established projection sites related with the function of sleep-wake controlled by A_2A_R neurons.

## MATERIALS AND METHODS

### ANIMALS

The adenosine A_2A_R-Cre mice have been well characterized in a previous study (Durieux et al., 2009, 2012). Adenosine A_2A_R-Cre and wild type (WT) mice were maintained in the Department of Molecular Behavioral Biology, Osaka Bioscience Institute, Osaka, Japan. Animals were given free access to food and water, and were housed in a room with an automatically controlled light-dark cycle (lights on at 07:00 and off at 19:00), an ambient temperature of 22 ± 0.5°C, and a relative humidity of 60 ± 2%. Animal Administrative Committee of Osaka Bioscience Institute approved all experimental procedures. All effort was made to minimize the numbers of animals used, and the suffering of the animals.

### BRAIN SURGERY

Adenosine A_2A_R-Cre and WT mice aged 18–20 weeks, weighing an average of 25 g, were stereotaxically injected with AAV-lox-stop-hrGFP following the procedure optimized by Chamberlin et al. (1998) and Baldwin et al. (2013). Under pentobarbital anesthesia (50 mg/kg, i.p.), the adenosine A_2A_R-Cre and WT mice were positioned in a stereotaxic apparatus, before a burr hole was made under aseptic conditions. A glass micropipette (10–20 μm outer tip diameter) containing the AAV-lox-Stop-hrGFP was connected to an air compression system. The glass micropipette was lowered into the brain at stereotaxic coordinates based on the mouse brain atlas (Paxinos and Franklin, 1997), viz, the AcbC (AP: +1.1 mm; ML: 1.0 mm; DV: 3.8 mm), the medial AcbSh (AP: +0.98 mm; ML: 0.5 mm; DV: 4.0 mm), and the ventral AcbSh (AP: +1.10 mm; ML: 1.0 mm; DV: 5.0 mm). The AAV-lox-Stop-hrGFP was delivered by slow pressure injection lasting 15 min to allow the tracer to diffuse into the brain. After leaving the pipette in the brain for an additional 10 min, the pipette was slowly retracted. Finally, a layer of gel-film was placed over the region of the craniotomy, and surgical staples were used to close the incision. Mice were then placed on a constant temperature plate and monitored during recovery. Mice were then returned to their home cage when fully awake.

### TISSUE PREPARATION

Four weeks after surgery, mice were deeply anesthetized with chloral hydrate (500 mg/kg, i. p.), and transcardially perfused with 0.9% saline followed by 4% paraformaldehyde (Sigma) in 0.1 M phosphate buffer (PB, pH 7.4). Brains were removed from the skull immediately after perfusion, post fixed in 4% paraformaldehyde at 4°C for 2 h, and transferred to 20% sucrose in 0.1 M PB. Serial sections of 30 μm were cut on a Leica freezing microtome in the coronal plane, collected in 0.1 M PB (pH 7.4), protected in a cryoprotectant solution, and stored at -20°C until further processing for immunohistochemistry.

### IMMUNOHISTOCHEMICAL STAINING

To identify A_2A_R neurons and their projections in various brain regions, hrGFP-expressing AAV was injected into the Acb. We had intended to use fluorescence microscopy to directly monitor the fluorescence of hrGFP in the brain sections. However, the hrGFP fluorescence in anterogradely labeled perikarya and axons of the neurons was too weak to be visualized. Brain sections of the transgenic mice were therefore stained with anti-hrGFP antibody to detect hrGFP at a higher sensitivity using both avidin–biotin complex (ABC) and 3, 3′-diaminobenzidine tetrahydrochloride (DAB; Vector Laboratories; cat. no. SK-4100).

For the ABC method, stored free-floating sections were washed in PBS for 15 min with three changes of buffer, and treated for 30 min in 3% H_2_O_2_ to quench endogenous peroxidase activity. After washing, the sections were blocked for non-specific binding of the secondary antibody in 5% normal goat serum (NGS; Vector Laboratories; cat. no. S-1000, lot no. X0815) and 0.3% Triton X-100 in PBS for 1 h at room temperature (RT). The sections were then incubated overnight at 4°C with hrGFP polyclonal rabbit antiserum (Stratagene; cat. no. 240142; lot no. 0830269; 1:30,000) followed by a biotinylated goat anti-rabbit IgG antibody (Vector Laboratories; cat. no. BA-1000; lot no. W2206), and ABC solution (Vectastain Elite ABC Kit; Vector Laboratories; cat. no. PK-4000; 1:1,000). After washing in PBS, the sections were visualized using DAB, washed in PBS, mounted on gelatin-coated slides, and air dried overnight. Some sections were also counter-stained with 0.1% cresyl violet. Finally, the sections were dehydrated in ascending alcohol concentrations, cleared in xylene, and cover slipped.

For immunofluorescence, the sections were blocked for non-specific binding of the secondary antibody in 5% NGS and 0.3% Triton X-100 in PBS for 1 h at RT. Sections were then incubated overnight at 4°C in rabbit anti-hrGFP antiserum (Stratagene; cat. no. 240142, lot no.0830269; 1:5,000) in 5% NGS (Vector, laboratories, cat. no. S-1000; lot no. X0815) with 0.3% Triton X-100 in PBS. Following incubation in primary antiserum, the sections were incubated with Alexa 488-conjugated anti-rabbit IgG (H + L) secondary antibody (Invitrogen, La Jolla, CA, USA; cat. no. A11034; lot no. 1073084; 1:500) for 1 h at RT. After washing in PBS, the sections were mounted on glass slides and coverslipped with Fluoromount-G^TM^ mounting medium (Southern Biotech; Catalog No, 0100-01). Negative controls omitting the primary antibody were carried out in all experiments, and did not show any immunoreactivity.

### MICROSCOPY AND CHARTING

The sections were observed and photographed on an Olympus IX71 microscope with fluorescent and bright-field optics. Adobe Photoshop CS2 was used for post-processing of digital photomicrographs and the schematic illustrations. The sharpness, color balance, and contrast were adjusted to obtain an optimal match of labeled cells or fibers against the background. Anatomical landmarks and abbreviations used in the present study were based on the Paxinos and Franklin mouse brain atlas (Paxinos and Franklin, 1997). High resolution fluorescent images of hrGFP were generated using a Leica SP2 confocal laser scanning microscope (Leica, Mannheim, Germany).

We evaluated the outlines of sections and major structures at low magnification (4×), and then mapped the profiles of the hrGFP immunoreactive (hrGFP-IR) perikarya and projection fibers under high magnification. Plotting of labeled cell bodies and fibers was done by Adobe Photoshop CS2. Using a method for semi-quantitative evaluation optimized by Gautron et al. (2010) and Bálint et al. (2011), the density of hrGFP-IR terminal networks was subjectively determined in six grades of fiber weight as follows: ++++, abundant; +++, numerous; ++, moderate; +, few; ±, sparse; and -, absent (**Table 1**). Processing and measurement of the varicosities/boutons were performed using image analysis Image J (National Institutes of Health, USA) as shown in **Table 1**.

**Table 1:** Distribution of hrGFP-positive varicosities and terminal networks following AAV-lox-Stop-hrGFP injections into the AcbC, mAcbSh, and vAcbSh.^1^

Regions	Acbc		mAcbSh		vAcbSh	
	Varicosities	Terminal networks	Varicosities	Terminal networks	Varicosities	Terminal networks
**Forebrain**
BST	–	–	81	+	–	–
BSTLP	267	++	–	–	–	–
VP	998	++++	80	++	197	+++
VDB	189	+	38	++	93	++
HDB	126	++	157	+	–	–
BMA	433	++	–	–	–	–
BLA	423	++	–	–	–	–
CPU	38	+	–	–	–	–
SI	272	+++	165	++	794	+++
LGP	187	+++	–	–	–	–
**Diencephalon**
LPO	212	+++	475	++	204	++
LHa	936	++++	244	++	215	+++
LHt	2023	++++	324	+++	571	+++
LHm	334	+++	155	++	772	+++
AHP	423	+++	–	–	–	–
Pe	138	±	–	–	–	–
DM	1228	+++	–	–	99	++
EP	33	+	–	–	–	–
VMHDM	822	++	–	–	–	–
PH	244	++	442	+++	203	++
VTM	47	+	–	–	–	–
DTM	64	±	–	–	–	–
STh	96	++	–	–	–	–
**Brainstem**
VTA	295	+++	39	+	119	+++
MnR	244	++	–	–	82	+++
DR	278	++	–	–	214	++
VLPAG	127	+	–	–	234	++
DRI	–	–	–	–	39	++
MPB	–	–	–	–	23	++
LC	–	–		–	12	+

1 ++++, Abundant; +++, numerous; ++, moderate; +, few; ±, sparse; –, absent. All abbreviations are defined in the abbreviation section.

## RESULTS

### INJECTION LOCATION AND MORPHOLOGY OF A_2A_R NEURONS

**FIGURE 1:**
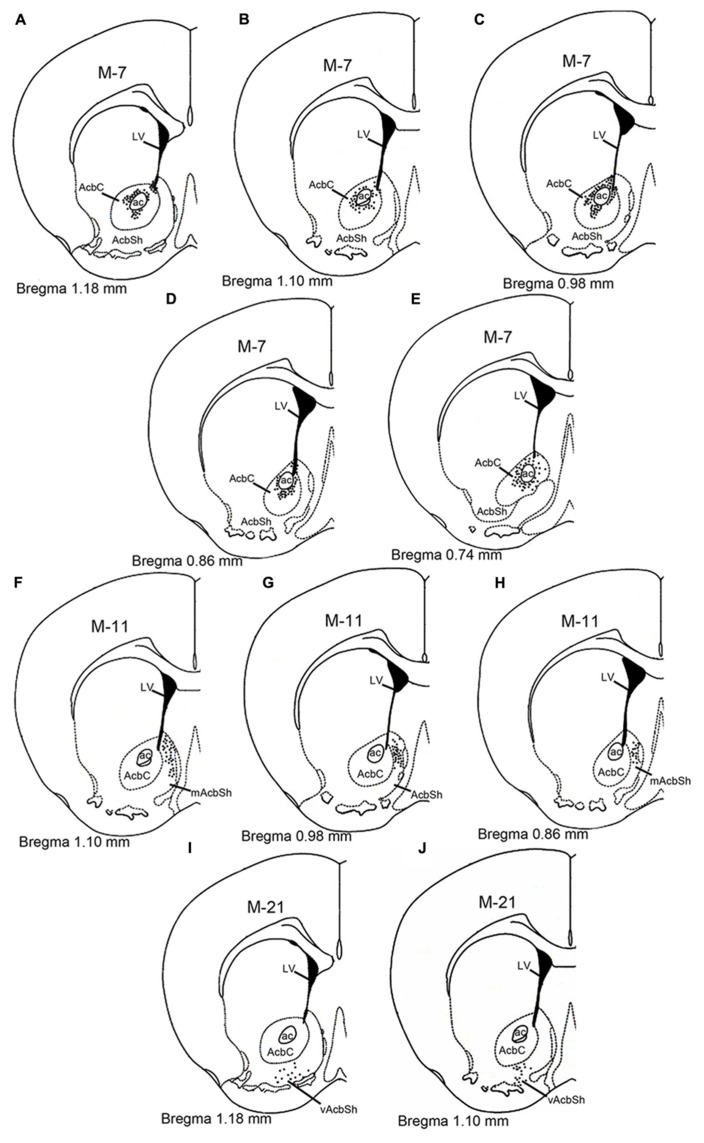
**Schematic diagram showing the AAV-lox-Stop-hrGFP injection sites in the nucleus accumbens**. The dotted areas indicate the injection locations in the core **(A–E)**, medial shell **(F–H)**, and ventral shell **(I–J)**. Each dark dot represents one labeled cell body. Abbreviations are as follows: ac, anterior commissural nucleus; AcbC: nucleus accumbens, core; AcbSh, nucleus accumbens, shell; mAcSh, medial AcbSh; vAcSh, ventral AcbSh; LV, lateral ventricle.

Visualized by either fluorescent or ABC techniques, injections of AAV-lox-Stop-hrGFP revealed small deposit sites in the Acb in 15 animals of the present study. Three typical injections affected the AcbC (M-7; **Figures 1A–E**), the medial AcbSh (M-11; **Figures 1F–H**), and ventral AcbSh (M-21; **Figures 1I,J**), and were chosen for the present analyses. The injection locations of three cases were schematically described in **Figure 1**. Three cases showed that hrGFP was almost exclusively confined to the AcbC, mAcbSh, and vAcbSh, respectively, except for the case (M-7) showed few expressiones outside the AcbC in neurons located in the caudate putamen (**Figure 1A**) and the dorsal part of AcbSh (**Figures 1A–D**).

**FIGURE 2:**
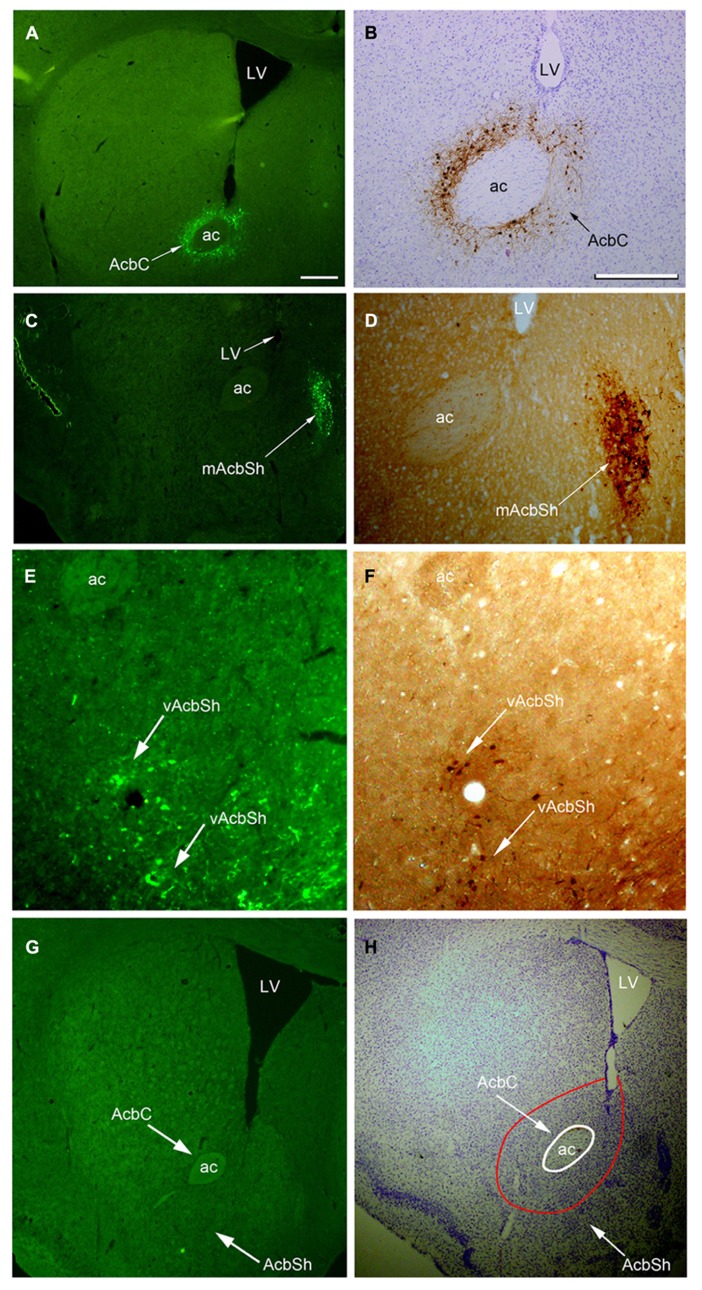
**Images of hrGFP immunoreactivity (hrGFP-IRc) showing examples of the AAV-lox-Stop-hrGFP injection sites. (A,B)** hrGFP-IRc in the Acb visualized by fluorescent and ABC techniques with Nissl-counterstaining. hrGFP-IRc staining, confirming the presence of the A_2A_R neurons, was deposited around the Acb and covered most of the core area (arrows) in two labeled adjacent sections of this example (M-7). **(C,D)** hrGFP-IRc staining, indicating A_2A_R neurons in the AcbSh, visualized by fluorescence and ABC techniques. Positive staining with hrGFP-IRc occupied the mAcbSh in **(C,D)** in two labeled adjacent sections of this case (M-11). **(E,F)** hrGFP-IRc staining, indicating A_2A_R neurons in the vAcbSh, visualized by fluorescence and ABC techniques. Positive staining with hrGFP-IRc occupied vAcbSh in **(E,F)** in two labeled adjacent sections in this case (M-21). **(G,H)** A brain section from the wild type mouse with fluorescent staining **(G)** and ABC techniques with Nissl-counterstaining **(H)** showing no hrGFP-IRc in Acb. Abbreviations are as follows: AC, anterior commissural nucleus; LV, lateral ventricle; AcbC, nucleus accumbens core; AcbSh, nucleus accumbens shell; mAcbSH, medial AcbSh; vAcbSh, ventral AcbSh. Scale bar = 500 μm in **(A)**; 100 μm in** (B)**; 500 μm in **(C)**; 100 μm in **(D)**; 100 μm in **(E)**; 100 μm in **(F)**; 500 μm in **(G);** 500 μm in **(H)**.

On the sections, the presence of hrGFP-IRc, indicating A_2A_R neurons, was observed as strong fluorescent or DAB reactive elements in clustered perikarya, dendrites, and proximal axons within both the AcbC and the AcbSh in adenosine A_2A_R-Cre mice (**Figures 2A–F**), but not in the WT mice (**Figures 2G,H**). Additionally, we analyzed the A_2A_R neuron cell bodies within the Acb and the distribution of the hrGFP-IR axonal fibers using the hrGFP-IRc antibody in samples from the three animals on typical serial sections of the brain from bregma +0.98 to -5.40 mm (**Figures 4–7**).

**FIGURE 3:**
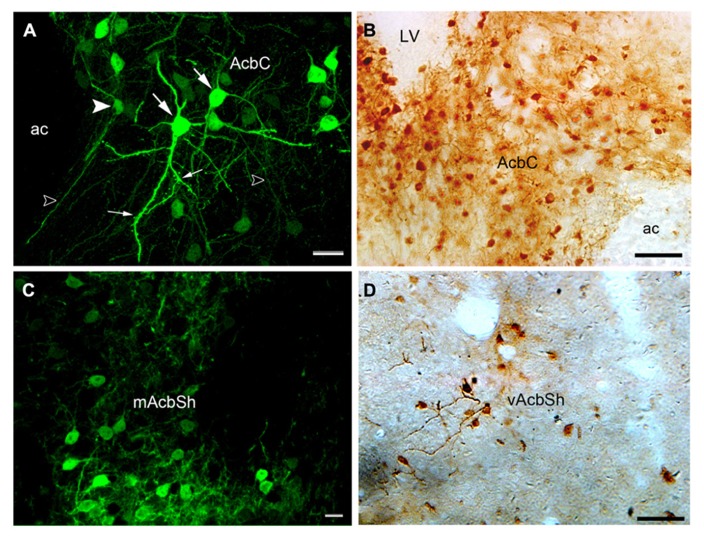
**Images showing hrGFP-IR-positive neurons in the Acb of the adenosine A_**2A**_R Cre mouse. (A,B)** The hrGFP-IR neurons in the core visualized by fluorescent and ABC techniques. Most of the perikarya in the core were observed as multipolar (large arrows) and bipolar (arrowhead) neurons. The dendrites extending from the perikarya were thick, and some of them had punctuate spines (small arrows). The axons were thin, straight fibers (opened arrows). **(C)** hrGFP-IR neurons in the mAcbSh visualized by fluorescent and ABC techniques. The neurons in this region were similar in shape to those in the core. **(D)** The neurons in the vAcbSh visualized by ABC techniques. AC, anterior commissural nucleus; LV, lateral ventricle; mAcbSh, medial AcbSh; vAcbSh, ventral AcbSh. Scale bar = 20 μm in **(A)**; 50 μm in **(B)**; 10 μm in **(C)**; 50 μm in **(D)**.

At high magnification (**Figures 3A–D**), hrGFP-IR neurons were observed as multipolar or bipolar neurons, and a higher density of the perikarya was observed in the lateral part of the core compared with the medial region. The perikarya of the neurons were mainly round or oval in shape, and measured approximately 10–15 μm in diameter. Dendrites extended from their perikarya and bifurcated in different directions. Most dendrites possessed puncta, indicating dendritic spines and the features of less-spiny neurons. hrGFP-IR axons were thin thread-like fibers that coursed straight off the Acb **Figure 3A**. These observations are consistent with the previous reports in rat models (Meredith et al., 1992).

### PROJECTIONS OF THE ACB A_2A_R NEURONS

**FIGURE 4:**
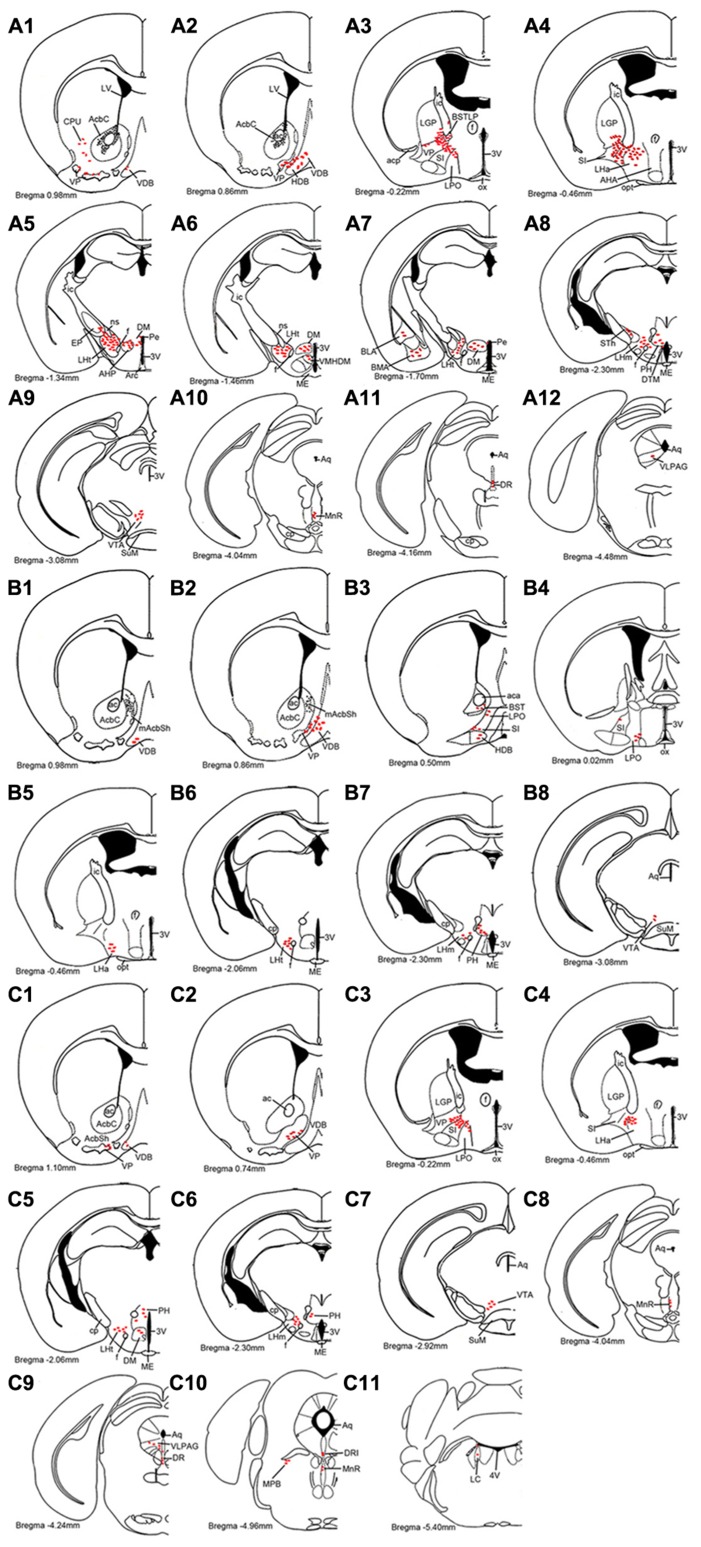
**Schematic drawings showing the distribution profiles of hrGFP-IR fibers and termini from the AcbC (A1–A12; M-7), medial AcbSh (B1–B8; M-11), and ventral AcbSh (C1–C11; M-21) injections**. The hrGFP-IR axons and terminals are indicated by red ellipse dots. The projections from the AcbC and ventral AcbSh extend more caudally than those from medial AcbSh. All abbreviations are defined in the abbreviation section.

**FIGURE 5:**
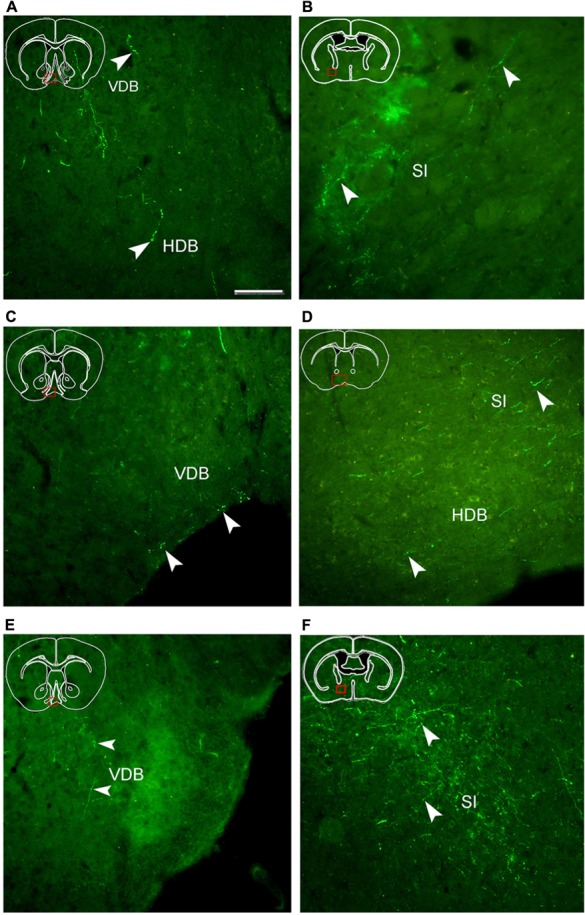
**Fluorescent images showing hrGFP-IR fibers and termini from the AcbC (A,B; M-7), medial AcbSh (C,D; M-11), and ventral AcbSh (E,F; M-21) injections**. Axons with varicosities invade the nucleus of the vertical limb of the diagonal band (VDB; **A,C,E**), the nucleus of the horizontal limb of the diagonal band (HDB; **A,D)**, and the substantia innominate (SI; B,D,F). (A,B) hrGFP-IR axons and termini in the VDB, the HDB, and the SI following the injection of AAV-lox-Stop-hrGFP into the AcbC. **(C,D)** hrGFP-IR axons and termini in the VDB, the HDB, and the SI following the injection of AAV-lox-Stop-hrGFP into the medial AcbSh. **(E,F)** hrGFP-IR axons and termini in the VDB and the SI following the injection of AAV-lox-Stop-hrGFP into the ventral AcbSh. Arrowheads indicate varicosities. Scale bar = 100 μm.

**FIGURE 6:**
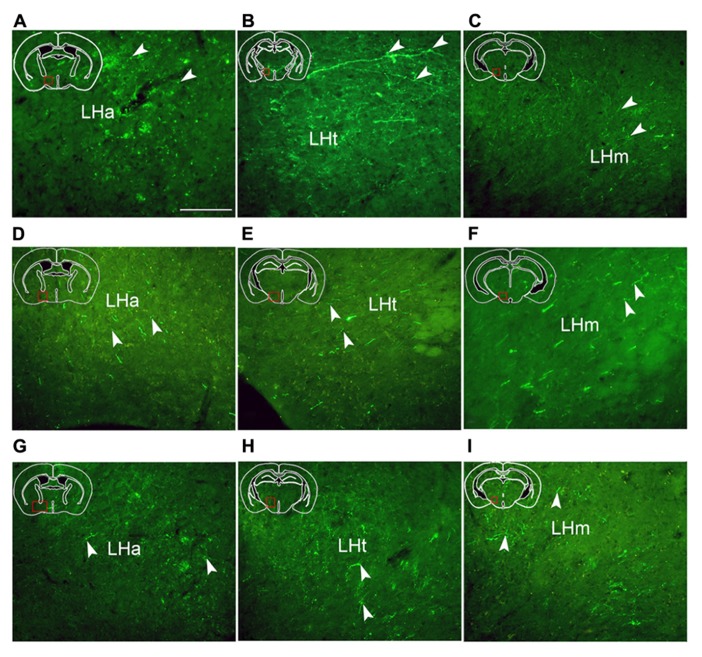
**Fluorescent images demonstrating hrGFP-IR axons and termini from the AcbC (A,B,C; M-7), the medial AcbSh **(D,E,F**; M-11), and the ventral AcbSh **(G,H,I**; M-21)**. Axons invade the anterior part of lateral hypothalamus (LHa), the tuberal part of lateral hypothalamus (LHt), and the mammillary part of lateral hypothalamus (LHm) after AcbC, medial AchSh, and ventral AcbSh injection, respectively. Arrowheads indicate varicosities. Scale bar = 100 μm.

**FIGURE 7:**
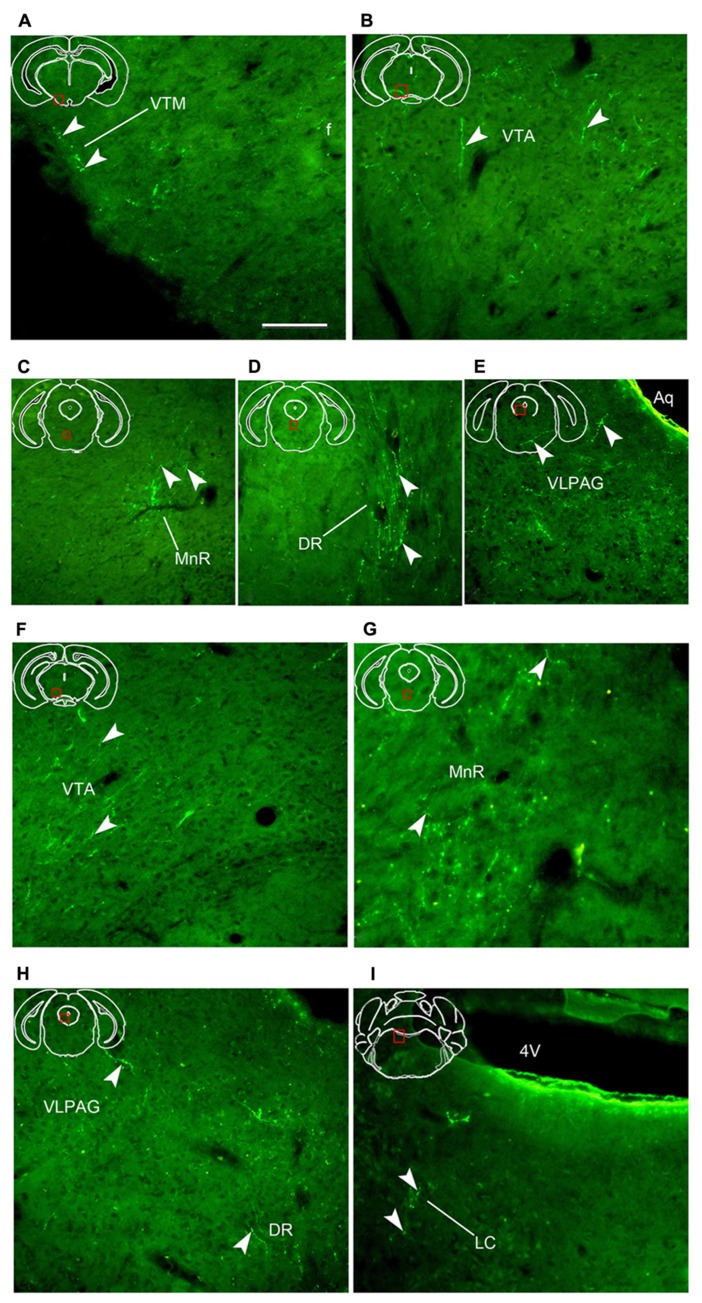
**Fluorescent images showing hrGFP-IR axons and termini from the AcbC (A–E; M-7) and ventral AcbSh (F–I; M-21) injections. (A–E)** Axons with varicosities invade the ventral tuberomammillary nucleus [**(A)**, VTM], the ventral tegmental area [**(B)**, VTA], the medial raphe nucleus [**(C)**, MnR], the dorsal raphe nucleus [**(D)**, DR], and the ventrolateral periaqueductal gray [**(E)**, VLPAG] after injection with AAV-lox-hrGFP into the AcbC (M-7). **(F–I)** Axons with varicosities invade the ventral tegmental area [**(F)**, VTA], the medial raphe nucleus [**(G)**, MnR], the dorsal raphe nucleus [**(H)**, DR], the ventrolateral periaqueductal gray [**(H)**, VLPAG], and the locus coeruleus [**(I)**, LC] following the injection of AAV-lox-hrGFP into the mAcbSh (M-22). Arrowheads indicate varicosities. Abbreviations: 4V, forth ventricle; Aq, aqueduct; f, fornix. Scale bar = 100 μm.

hrGFP-IR axons from Acb A_2A_R neurons were distributed in the ipsilateral hemisphere to the injection site. The distribution patterns of hrGFP-IR axons after injection into the AcbC, medial AcbSh, and ventral AcbSh are demonstrated schematically in **Figure 4**, respectively. Finally, we depict the brain areas related to sleep-arousal in detail in **Figures 5–7**. The relative density of hrGFP-IR axons and terminals in each targeted area is summarized in **Table 1**.

### FORBRAIN PROJECTIONS

Following injection into the AcbC (M-7), hrGFP-IR axons extended laterally from the injection site into the adjacent ventral portion of the caudate putamen (CPu; 38 boutons/varicosities), ventrally into the ventral pallidum (VP; 998 boutons/varicosities), and medially into the caudal portion of the nucleus of the vertical limb of the diagonal band (VDB; 189 boutons/varicosities; **Figure 4A1**). At a more posterior level, some axons with varicosities proceeded longitudinally in the ventromedial portion of the anterior commissure, throughout the caudal AcbSh into the medial VP, the caudal VDB, and the rostral nucleus of the horizontal limb of the diagonal band (HDB; 126 boutons/varicosities; **Figure 4A2**). Axons were coursing in a wavy form and varicosities were appearing big round and small ellipse (**Figure 5A**). The mean diameter of varicosities in the HDB (2.103 μm) was bigger than in the VDB (1.939 μm). At the caudal level of the optic chiasm, a dense plexus of axons with numerous varicosities were observed in the dorsal portion of the VP, which turned dorsally into the ventral part of the lateral globus pallidus (LGP; 187 boutons/varicosities), ventromedially to enter the substantia innominata (SI; 272 boutons/varicosities), and medially into the sub-regions of the bed nucleus of the stria terminalis (BST; **Figure 4A3**). More caudally, a number of axons were still found in the ventral LGP and the SI (**Figure 4A4**), and hrGFP-IR-positive axons formed small ellipse varicosities (1.841 μm mean diameter) within the SI and shifted medially into the rostral portion of the anterior lateral hypothalamus (LHa; **Figures 4A4** and **5B**). Injection in to the AcbC therefore resulted in more anterograde labeling in the VP than injections into the medial or ventral AcbSh (**Table 1**). In addition, moderate projections were observed in the anterior part of basomedial amygdaloid nucleus (BMA) and the anterior part of basolateral amygdaloid nucleus (BLA; **Figure 4A7**), visualized as diencephalic projections.

After the medial AcbSh injection (M-11), hrGFP-IR axons spread ventromedially from the injection site into the adjacent caudal VDB (38 boutons/varicosities; **Figures 4B1** and **5C**). Approximately 0.12 mm caudally from the injection site, axons were observed in the dorsomedial portion of the VP and the lateral margin of the caudal VDB (**Figure 4B2**). More distally, labeled ventrally oriented axons passed below the anterior commissure into the dorsolateral portion of the rostral lateral pre-optic area (LPO), the ventro-lateral portion of the rostral SI, and the HDB (**Figure 4B3**). Labeled fibers also formed small round and ellipse varicosities within the SI and HDB (**Figure 5D**). Injection in to the medial AcbSh therefore generated increased labeling in the VDB compared with injection into the AcbC (**Table 1**).

After ventral AcbSh injection (M-21), hrGFP-IR axons projected ventrally to the VP, and medially to the lateral border of the VDB (93 boutons/varicosities; **Figure 4C1**). Within the VDB, fibers, and terminals bearing few small ellipse varicosities were observed (**Figure 5E**). At the level of the caudal VDB, numerous stained fibers, and terminals were seen in the ventromedial VP (**Figure 4C2**). At the level of the caudal optic chiasm, hrGFP-IR axons formed a number of fascicles in the ventral portion of the VP, which spread ventrally into the dorsal portion of the SI (**Figure 4C3**). Within the SI, wavy hrGFP-IR axons appeared with small round varicosities (794 boutons/varicosities), sent some terminals to the SI (**Figure 5F**), and further extended medially into the dorsal portion of the adjacent LPO (**Figure 4C3**). More caudally, moderate hrGFP-IR axons with varicosities were still observed in the dorsomedial portion of the SI (**Figure 4C4**). Injection into the ventral AchSh therefore generated more hrGFP-IR axons and terminals in the SI than medial AcbSh injection (**Table 1**.

### DIENCEPHALIC PROJECTIONS

hrGFP-IR-stained axons from the AcbC (M-7), medial AcbSh (M-11), and ventral AcbSh (M-21) were observed in the LPO (**Figures 4A3,B3,4,C3**), and in the lateral hypothalamus (LH; **Figures 4A4–8,B5–7,C4–6**). The densest projections and the biggest varicosity were found in the tuberal part of the lateral hypothalamus (LHt; 2023 boutons/varicosities and 4.837 μm), originating in the core region (**Figures 4A5,6** and **6B**). In the rostral LHa and caudal LHt, numerous varicosity-bearing fibers and terminals were observed (**Figures 6A,B**). In mice that had been injected in the ventral AcbSh, numerous axons with moderate varicosities were also found in the LH (**Figures 4C4–6** and **6G–I**). However, these projection axons bearing less varicosities were fewer in number in mice injected into the medial AcbSh (**Figures 4B5–7** and **6D**–**F**; **Table 1**).

Following AcbC injection (M-7), hrGFP-IR axons were located in the dorsal portion of the LPO (212 boutons/varicosities) at the level of optic chiasm (**Figure 4A3**), and the dorsal LHa (936 boutons/varivosities) at the level of optic tract (**Figure 4A4**). Within the LHa, varicosities formed groups and mainly showed small round (**Figure 6A**). Axons from the medial AcbSh projected in to the dorsolateral LPO beneath the anterior commissural nucleus (ac; **Figure 4B3**), the ventromedial LPO at the level of optic chiasm (**Figure 4B4**), and the ventral LHa. Within the LHa, varicosities were sparsely distributed and showed small round and ellipse (**Figure 6D**). However, after ventral AcbSh injection, hrGFP-IR axons were found in the dorsal part of LPO at the level of optic chiasm (**Figure 4C3**), and the dorsolateral portion of LHa (**Figure 4C4**). The mean diameter (1.575 μm) of the varicosities in the LHa following injection into ventral AcbSh was smaller than the mean diameter of both varicosities of the AcbC (2.085 μm) and mAcbSh (1.838 μm).

Throughout the length of the hypothalamus, hrGFP-IR axons extended around the LH after AcbC injection (M-7). At the level of the rostral LHt, the fibers traversed along the dorsal portion of the fornix, extended medially into the AHP (423 boutons/varicosities), and the periventricular hypothalamic zone, and spread dorsolaterally to reach the ventromedial part of the entopeduncular nucleus (EP; 33 boutons/varicosities; **Figures 4A5** and **6B**). The nerve plexus then spread out medially into the dorsomedial hypothalamic nucleus (DM; 1228 boutons/varicosities), and the dorsomedial portion of the ventromedial hypothalamic nucleus (VMHDM; 822 boutons/varicosities; **Figure 4A6**). Caudally to this, some axons extended laterally through the sublenticular region to the BMA and BLA (**Figure 4A7**). At the rostral mammillary part of lateral hypothalamus (LHm), axons spread dorsomedially into the posterior hypothalamic area (PH), and the dorsal tuberomammillary nucleus (DTM; **Figure 4A8**). In addition, some hrGFP-IR axons were found to run along the medial margin of the subthalamic nucleus (STh), projecting terminals into the STh (96 boutons/varicosities; **Figure 4A8**). Adjacent to this, there were also some projections to the ventral tuberomammillary nucleus (VTM; **Figure 7A**).

After medial AcbSh injection (M-11), hrGFP-IR axons only extended dorsomedially into the PH (442boutons/varicosities) at the level of the rostral LHm (**Figure 4B7**). In contrast, hrGFP-IR axons spread out medially or dorsomedially into the DM (99 boutons/varicosities) or the PH (203 boutons/varicosities) at the level of the caudal LHt after ventral AcbSh injection (**Figure 4C5**).

### BRAINSTEM PROJECTIONS

A_2A_R neurons of the AcbC (M-7), medial AcbSh (M-11), and ventral AcbSh (M-21) were found to project to similar areas of the ventral tegmental area (VTA; **Figures 4A9,B8,C7**). In case of AcbC, hrGFP-IR fibers were mainly located in the dorsal part of the VTA, formed numerous varicosities (295 boutons/varicosities), and had short, straight processes (**Figure 7B**). For ventral AcbSh injection, hrGFP-IR fibers bearing moderate varicosities were observed mainly in the ventral portion of the VTA (119 boutons/varicosities), and also processed shortly and straightly (**Figure 7F**). In case of medial AcbSh injection, however, few projections were found in the medial part of the VTA (39 boutons/varicosities; **Figure 4B8**), and so their processes could not be followed. The varicosities mean diameter in the VTA following injection of AcbC, mAcbSh, and vAcbSh was 1.801, 1.67, and 1.635 μm, respectively.

After AcbC injection (M-7), the axons shifted dorsomedially through the lateral margin of the rostral median raphe nucleus (MnR; **Figures 4A10** and **7C**), while some axons continued to the ventral portion of the dorsal raphe nucleus (DR; **Figures 4A11** and **7D**), and into the ventrolateral periaqueductal gray (VLPAG; **Figures 4A12** and **7E**). Within the DR, varicosities showed medium round and vertical distribution. The varicosities in the VLPAG were small round or ellipse and transverse distribution.

In case of the ventral AcbSh injection (M-21), the axons traveled dorsally along the lateral border of the MnR (**Figures 4C8** and **7G**), continuing to the caudal DR (**Figures 4C9** and **7H**). The remaining axons then entered the VLPAG (**Figure 4C9**), and ran wavy (**Figure 7H**). More caudally, hrGFP-IR axons bearing few varicosities were found in the caudal MnR (82 boutons/varicosities), and turned into the ventral part of dorsal raphe nucleus (DRI; **Figure 4C10**). Additionally, moderate fibers with few varicosities and termini were found in the medial parabrachial nucleus (MPB; **Figure 4C10**). Finally, few axons were traced into the locus coeruleus (LC) and formed sparse swellings (12 boutons/varicosities) in the rostral forth ventricle (**Figures 4C11** and **7I**), but nothing further was observed. The boutons in the MnR mainly presented medium round (**Figure 7G**), whereas in the VLPAG and DR they were small round or ellipse (**Figure 7H**).

## DISCUSSION

Because of the important physiological, pathological, and pharmacological functions of the A_2A_R in Acb, the present study mapped the projections of A_2A_R neurons in various brain regions in A_2A_R-Cre mice using a tracer of AAV-lox-Stop-hrGFP. Our data revealed significant differences in the areas innervated by the AcbC, medial AcbSh, and ventral AcbSh. The projection sites of Acb A_2A_R neurons were summarized, allowing the formation of a neuromorphological basis to better understand the functions of the Acb A_2A_R, and especially its role in regulating sleep-wake behavior.

### TECHNICAL CONSIDERATIONS

Efferent projections of Acb have previously been studied in great detail using conventional neuroanatomical methods (Heimer et al., 1991; Zahm and Heimer, 1993; Usuda et al., 1998). Systematic tracing of axonal projections from specific neurons remains challenging using conventional tracking methods. However, novel technologies allowing individual neurons to be selectively traced have been developed. First, DeFalco et al. (2001) used virus-assisted mapping to identify leptin receptor-expressing neuronal afferent projections. Additional work developed transgenic mouse models expressing a fusion protein of the C-terminal fragment of tetanus toxin and GFP specifically in orexin neurons, allowing visualization of orexin neurons (Sakurai et al., 2005). Recently, Hnasko et al. (2012) used approach similar to that used here to trace the projection of glutamate neurons in the ventral tegmental area using a conditional channel rhodpsin-expressing AAV (AAV-EF1a-DIO-ChR2-mCherry). Although these approaches differ from the approach taken here, these methods provide a useful reference for tracing other axonal projections of selected neurons.

The approach used in the present study was previously described and optimized by Gautron et al. (2010). Additionally, the transgenic mouse model used here has been well characterized in a previous report (Durieux et al., 2009, 2012). Because of the inherent difficulty of conventional approaches for the tracing of specific neurons, we used a novel approach based on the conditional expression of hrGFP. Briefly, AAV was stereotaxically injected into the AcbC and AcbSh of mice expressing Cre recombinase under the control of the adenosine A_2A_R promoter (Durieux et al., 2009, 2012). Consequently, mice exhibited robust hrGFP expression specifically in Acb A_2A_R neurons after injection. Importantly, hrGFP was efficiently transported in axonal projections to brain regions innervated by the Acb A_2A_R neurons.

### ACB A_2A_R NEURONS PROJECTIONS AND COMPARISONS WITH PREVIOUS STUDIES

For the AcbC, the main forebrain projections occur to the BST sub-regions, the VP, the CPu, the diagonal band nucleus, the SI, the LGP, the BLA, and the BMA, while the primary diencephalon targets are the LPO, the LH, the AHP, the DM, the VMHDM, the PH, the TMN, and the STh. Finally, the major brain stem projections are to the VTA, the MnR, the DR, and the VLPAG. In case of medial AcbSh, the major forebrain projection sites are the BST, the VP, the diagonal band nucleus, and the SI. In contrast, the projected areas from the diencephalon are the LPO, the LH, and the PH, while in the brainstem, the medial AcbSh only projects to the VTA. When projections from ventral AcbSh are considered, the forebrain targets are the diagonal band nucleus, the VP, and the SI, while the main diencephalon sites are the LPO, the LH, the DM, and the PH. Finally the primary brainstem targets are the VTA, the MnR, the DR, the VLPG, the DRI, the MPB, and LC.

As described above, A_2A_R neurons from Acb sub-regions jointly project to the VP. Although the projections of Acb A_2A_R neurons to the VP have been well described in rats (Mingote et al., 2008), little research has been carried out to characterize the overall projection patterns of Acb A_2A_R neurons. The most thorough analysis of Acb efferent projections was carried out in an early study using an autoradiographic fiber-tracing method (Nauta et al., 1978) and BDA tracer (Usuda et al., 1998). Additional studies, using PHA-L and BDA, described Acb projections in the cat (Troiano and Siegel, 1978; Groenewegen and Russchen, 1984), rhesus monkey (Haber et al., 1990), rat (Heimer et al., 1991; Zahm and Heimer, 1993), and chicken (Bálint et al., 2011).

In general, our findings are consistent with the previous studies, with the exception that some reports (Heimer et al., 1991; Usuda et al., 1998) revealed fairly widespread projections from the middle to caudal Acb to other sites, including the substantia nigra pars compacta (SNC), retrorubral area (RR), pedunculopontine tegmental nucleus (PPTg), substantia nigra pars reticulate (SNr), substantia nigra pars lateralis (SNI), paramedian raphe nucleus (PMR). We did not observe axons in any of these structures, which may be explained by the absence of projections from Acb neurons expressing A_2A_Rs. In the ventral striatum, GABAergic efferent neurons can be characterized into two major classes: GABAergic enkephalinergic, and GABAergic dynorphinergic neurons (Ferré et al., 2007). In addition to these two groups, large cholinergic interneurons and GABAergic striatal interneurons also exist (Rosin et al., 2003). Adenosine A_2A_Rs are found in the dendritic spines of GABAergic enkephalinergic neurons or in the glutamatergic terminals (Ferré et al., 2007; Schiffmann et al., 2007). It is plausible that efferent enkephalinergic and dynorphinergic Acb neurons project differentially to other brain sites. Moreover, absence of projection in these regions could be due to insufficient coverage of employed AAVs to whole caudal Acb or less sensitivity of the technique in the present study than conventional tracers.

The present mapping showed that AcbC projects to the lateral nucleus of amygdala, consistent with previous findings (Nauta et al., 1978). AcbC injection results in the projection of axons into the periventricular hypothalamic zones, the dorsal hypothalamus area, the PH, the mammillary body, and the EP. However, unlike this study, we demonstrated that AcbC also projects some axons to the BLA. In addition, we also observed that caudal AcbC injection results in projections to the BMA and the DR, none of which had been previously described. A previous study revealed that the medial AcbSh projects to the VP, the BST, the LPO, the LH, and VTA (Usuda et al., 1998). We also observed numerous projections to the VDB and the PH, and few to the HDB. Consistent with the present data, Usuda et al. (1998) depicted ventral AcbSh projections to the VP, the SI, the LPO, the LH, the VTA, the PAG, the PB, and the LC. Furthering these observations, we also detected numerous axons projecting to the HDB and the MnR, and to a lesser extent to the DR, and DRI. Although the reason for this discrepancy could be explained by the different techniques used, or by differences in species of the animals used in each study, we also suggest other important considerations in the following aspects: (1) specific injection site results in the different projections, which is not an exception for this method. For example, AcbC injection in the present study covers the greatest extent core area, although not whole, while the tracer is separately located in a restricted position in the previous studies; (2) few off-site infected neurons, if any, may send projections to those new findings. (3) The possibility of tracer uptake by fibers of passage also lead to those projections; (4) few employed AAVs possibly may be from transport to second-order neurons, which send fibers to those regions. However, the above-mentioned problems are inherent in most tracing approaches but are not specific to our study. In spite of these differences, it is reassuring to see that these techniques identify many of the same efferent projections, but additional studies should be carried out to further explain the discrepancies.

### FUNCTIONAL IMPLICATIONS

The major functional implication for the present results is that the Acb adenosine A_2A_R may have functions in the brain sites receiving axon projections from A_2A_R neurons. A comparison of the axon distributions with the prior studies (Nauta et al., 1978; Heimer et al., 1991; Usuda et al., 1998) may support the current paradigms of the related functions of Acb adenosine A_2A_Rs. However, the problem emerges as to whether there is a unified understanding of how anatomical considerations and fiber projections regulate behavioral or physiological processes. The established functional roles of the region most heavily innervated by the A_2A_R neurons suggests that Acb adenosine A_2A_Rs are involved in the regulation of sleep (Huang et al., 2007, 2011; Lazarus et al., 2011, 2012, 2013).

A compelling framework that Acb and A_2A_R modulate wakefulness and sleep comes from the studies of Acb lesions and blocking adenosine A_2A_R. Acb lesions lead to a significant increase in the amount of wakefulness (Qiu et al., 2010), while ablating both AcbC and AcbSh increases wakefulness but the core lesion plays a more important role in sleep-wake regulation (Qiu et al., 2012). Adenosine A_2A_Rs are abundantly expressed in the Acb (Rosin et al., 1998). Recent studies have revealed that A_2A_Rs play a crucial role in the regulation of sleep (Huang et al., 2005; Lazarus et al., 2012, 2013), and that AcbSh A_2A_R mediates the arousal effect of caffeine, a non-specific adenosine A1R and A_2A_R antagonist (Lazarus et al., 2011). However, the neural pathways exerting these effects have not yet been identified. The sites identified in the current work provide some insight, since Acb A_2A_R neurons give off the densest projection to the LH (containing orexin/hypocretin) which can activate the noradrenergic cells of the LC, the dopaminergic cells of the VTA, the serotonergic cells of the DR, and the histaminergic cells of the tuberomammillary nucleus (TMN), all of which cause arousal from sleep (Sakurai, 2007). In addition, Acb A_2A_R neurons project to the TMN, the VTA, the DR, the VLPAG, and the LC. As a group, these structures form the ascending arousal system, and fire fastest during wakefulness (Saper et al., 2005). Other innervated sites are located in the basal forebrain, including the VDB, the HDB, and the SI, and these neurons augment the ascending arousal system (Saper et al., 2005), or project to the wake-regulating lateral hypothalamus (Henny and Jones, 2006). A consistent observation is that adenosine A_2A_R in the Acb is almost entirely expressed in striatopallidal projection neurons in rodents as well as in primates (Schiffmann et al., 1991a,b; Fink et al., 1992). This suggests that A_2A_R neurons are inhibitory GABAergic projection neurons. Taken together, our data reveal relationships between Acb A_2A_R and the areas related with wakefulness, and suggest the possible neural pathways mediating the regulation of Acb A_2A_Rs in promoting sleep. Furthermore, injection to the AcbC, medial AcbSh, and ventral AcbSh projects to the above-mentioned sites as well as different sites related to wakefulness and sleep. We therefore suggest various candidate sub-regions in the Acb regulating sleep. Based on our data, the best candidates for the effect of AcbC adenosine A_2A_R on sleep would be basal forebrain (including VDB, HDB, and SI), orexin field, VTM, VTA, dorsal raphe, and VLPAG, for the medial AcbSh would be basal forebrain (including VDB, HDB, and SI), orexin field, VTA, and for the ventral AcbSh the basal forebrain (including VDB and SI), orexin field, VTA, DR, VLPAG, and LC. Indeed, the recent study reveals that GABAergic medium-sized spiny neurons in the AcbSh have marked differences in the receptor expression pattern, which activate the different signaling pathways and play the corresponding functions (Gangarossa et al., 2013). Future studies should therefore be directed to better understand whether characteristic synapses exist between Acb axons of A_2A_R neurons and these innervated sites.

## CONCLUSION

In summary, the present study specifically demonstrated the projections of Acb A_2A_R neurons by means of an injection of hrGFP-expressing AAV as a tracer in A_2A_R-Cre transgenic mouse. The results reveal that Acb A_2A_R neurons distribute their axons to several brain fields. As a consequence of these findings, the present results provide an anatomical basis for the physiologic, pathologic, and pharmacologic implications of Acb A_2A_R neurons. In particular, the present studies provide important data from which further studies can be designed to better understand the potential role of Acb A_2A_Rs in the regulation of sleep.

## Conflict of Interest Statement

The authors declare that the research was conducted in the absence of any commercial or financial relationships that could be construed as a potential conflict of interest.

## Author Contributions

Jian-Ping Zhang and Qi Xu have contributed equally to this work. All authors had full access to all the results in the study and take responsibility for the integrity of the results. Jian-Ping Zhang and Qi Xu were involved in studying concept and design, acquisition and analysis of data, and writing the article. Rui-Xi Li, Zhi-Li Huang, Michael Lazarus, and Yoshihiro Urade took part in the experimental design, helped in the analysis of the results and wrote the article. Serge N. Schiffmann and Alban de Kerchove d’Exaerde provided A_2A_R Cre mice. Wei-Min Qu was involved in designing the experiments and analysis of data.

## ABBREVIATIONS

3V: 3rd ventricle
4V: 4th ventricle
Ac: anterior commissural nucleus
Aca: anterior commissure anterior part
Acb: accumbens nucleus
AcbC: accumbens nucleus core
AcbSh: accumbens nucleus shell
acp: anterior commissure posterior
AHA: anterior hypothalamic area anterior part
AHP: anterior hypothalamic area posterior part
Aq: aqueduct
Arc: arcuate hypothalamic nucleus
BLA: basolateral amygdaloid nucleus anterior part
BMA: basomedial amygdaloid nucleus anterior part
BST: bed nucleus of the stria terminalis
BSTLP: bed nucleus of the stria terminalis lateral division posterior part
cp: cerebral peduncle basal part
CPu: caudate putamen
D3V: dorsal 3rd ventricle
DM: dorsomedial hypothalamic nucleus
DR: dorsal raphe nucleus
DRI: dorsal raphe nucleus ventral part
DTM: dorsal tuberomammillary nucleus
EP: entopeduncular nucleus
f: fornix
HDB: nucleus of the horizontal limb of the diagonal band
iC: internal capsule
LC: locus coeruleus
LGP: lateral globus pallidus
LH: lateral hypothalamus
LHa: anterior part of lateral hypothalamus
LHM: mammillary part of lateral hypothalamus
LHt: tuberal part of lateral hypothalamus
LPO: lateral preoptic area
LV: lateral ventricle
ME: median eminence
MnR: median raphe nucleus
MnR: nucleus of the vertical limb of the diagonal band
MPB: medial parabrachial nucleus
ns: nigrostriatal bundle
opt: optic tract
ox: optic chiasm
Pe: periventricular hypothalamic nucleus
PH: posterior hypothalamic area
SI: substantia innominata
STh: subthalamic nucleus
SuM: supramammillary nucleus
VDB: ventrolateral periaqueductal gray
VLPAG: ventromedial hypothalamic nucleus dorsomedial part
VMHDM: ventral pallidum
VP: ventral tegmental area
VTA: ventral tuberomammillary nucleus.
